# In the Right Light: Photodynamic Inactivation of Microorganisms Using a LED-Based Illumination Device Tailored for the Antimicrobial Application

**DOI:** 10.3390/antibiotics9010013

**Published:** 2019-12-30

**Authors:** Martina Hasenleitner, Kristjan Plaetzer

**Affiliations:** Laboratory of Photodynamic Inactivation of Microorganisms, Department of Biosciences, University of Salzburg, Hellbrunnerstr. 34, 5020 Salzburg, Austria

**Keywords:** Photodynamic Inactivation, photosensitizers, natural substances, light source, light emitting diode

## Abstract

Drug-resistant bacteria threaten the health of people world-wide and cause high costs to their health systems. According to Scientific American, the number of regrettable fatalities due to the bacteria that are resistant to conventional antibiotics will sum up to 300 million until 2050 if the problem is not tackled immediately. Photodynamic Inactivation (PDI) has proven effective against microorganisms irrespective of their resistance to conventional treatment, but for the translation into clinical practice, economic, homogenous and powerful light sources holding approval as medical devices are needed. In this study we present two novel light emitting diode (LED)-based lamps (Repuls7PDI-red and Repuls7PDI-blue) tailored for application in PDI and demonstrate their photodynamic efficiency upon using either methylene blue (MB), a photoactive compound widely used in PDI, or Sodium Magnesium Chlorophyllin (CHL), a water-soluble derivative of chlorophyll, which holds approval as food additive E140, against bacteria and fungi. Gram+ *Staphylococcus aureus*, Gram− *Escherichia coli* and the yeast *Candida albicans* serve as model systems. Repuls7PDI-red emits a wavelength of 635 nm and an intensity of 27.6 ± 2.4 mW·cm^−2^ at a distance of 13.5 cm between the light source and the target, while the Repuls7PDI-blue allows an exposure at 433 nm (within the range of violet light) (6.4 ± 0.5 mW·cm^−2^ at 13.5 cm). Methylene blue was photoactivated with the Repuls7PDI-red at 635 nm (25.6 J·cm^−2^) and allows for photokilling of *E. coli* by more than 6 log_10_ steps at a concentration of 10 µM MB. Using equal parameters, more than 99.99999% of *S. aureus* (20 µM MB) and 99.99% of *C. albicans* (50 µM MB) were killed. If blue light (Repuls7PDI-blue, 433 nm, 6.6 J·cm^2^) is used to trigger the production of reactive oxygen species (ROS), a photoinactivation of *S. aureus* (5 µM CHL, CFU reduction > 7 log_10_) and *C. albicans* (>7 log_10_) below the detection limit is achieved. PDI based on CHL (10 µM) using red light activation reduces the number of viable *S. aureus* by more than 6 log_10_. Our data prove that both LED-based light sources are applicable for Photodynamic Inactivation. Their easy-to-use concept, high light output and well-defined wavelength might facilitate the translation of PDI into clinical practice.

## 1. Introduction

Since their discovery about one hundred years ago, antibiotics have become the bedrock of modern medicine [1]. By principle, conventional antibiotics feature a single mode of action, which enables microorganisms to develop resistance strategies. As a result, the number of untreatable strains due to resistance to one or many drugs is constantly increasing. The omnipresence of antibiotics in the human ecosystem fuels bacterial resistance and threatens to eliminate antibiotics as effective drugs for therapeutic use in humans. 

Being aware of the fact that the pipelines for development of new antibiotics are about to dry out, health organizations such as the World Health Organization (WHO) urge the rapid establishment of novel and alternative antimicrobial approaches [2].

Photodynamic Inactivation utilizes the photosensitizer-mediated and light-induced overproduction of reactive oxygen species (ROS) to kill target microorganisms irrespective of their resistance to conventional treatment. As the generated ROS attack cellular structures in a random manner, the mode of action is unspecific, and therefore the rapid development of resistance against PDI very unlikely. The procedure is characterized by a simple but effective treatment protocol: in the first step a photoactive, non-toxic substance, the so-called photosensitizer (PS), is applied to the local bacterial infection. During short incubation—usually the drug to light interval is less than 15 min—the PS is absorbed by the bacterial cells or interacts with their cell wall. This is followed by an exposure of the target with visible light of an appropriate wavelength, which excites the PS in an activated triplet state. Transfer of energy or electrons to molecular oxygen leads to massive production of ROS. As one molecule of the photoactive compound can photocatalyze many entities of reactive oxygen, the effective concentration of the photosensitizers in the target cells may be relatively low. In addition, the diffusion of singlet oxygen is limited to 30 nm, which restricts the generation of cellular damage to the proximity of the photosensitizer [3].

Since the re-discovery of Photodynamic Inactivation more than twenty years ago, photosensitizers successfully employed in Photodynamic Therapy (PDT) against cancer have been tested for their application against microorganisms. Other compounds with properties optimized for fighting bacteria and fungi were synthesized. As a result, a wide spectrum of photosensitizers readily exist from the classes of phenothiazinium dyes [4], porphyrins [5], chlorins, phthalocyanines and even natural substances, such as chlorophyllin [6], curcumin or hypericin [4], are ready for their testing in clinical trials.

With PDI being a local treatment of usually larger areas, the development of light sources optimized for the antimicrobial application was neglected. In Photodynamic Therapy against cancer, lasers are widely used as illumination devices, as they can be easily coupled to light fibers to activate the photosensitizer present in tumors inside the human body (e.g., in the gastrointestinal tract or bladder). However, lasers are relatively expensive, and require additional optical systems, such as a light guide, to widen the beam. Furthermore, they usually require maintenance and special training of users is needed for safe operation. Non-coherent light sources such as tungsten filament-, quartz halogen-, xenon arc-, metal halide- and phosphor-coated sodium lamps are used to treat larger areas in the treatment of dermatological cancers or pre-cancers (e.g., actinic keratoses, [7]). In contrast to lasers these lamps emit a wide wavelength spectrum requiring filter systems to avoid the significant heating of the medium or tissue. The Waldmann PDT 1200 lamp (Waldmann Medizintechnik, Villingen-Schwenningen, Germany), a light source still widely used by dermatologists, is a broadband, non-coherent, metal halide light device that uses optical filters to remove ultraviolet and infrared components. It emits the wavelength range 570–730 nm (yellow to orange to red) without the ability to select a wider or narrower wavelength band. However, as a single 1200 W lamp is used in the device, the light field of this lamp has been reported to be rather inhomogeneous [8]. To overcome the limitations of lasers and non-coherent light sources, arrays based on light emitting diodes (LEDs) were developed [9,10]. They provide a high light output with low heat production, emit a limited wavelength range (usually below ±10 nm of the dominant wavelength), and offer a homogenous spatial light distribution if based on many individual LEDs [7]. 

Still many research groups in PDI use home-made LED light sources [11,12], which makes comparison of data between the individual studies difficult, and more seriously, impedes the translation of PDI into clinical practice, as doctors require simple to use, safe and approved illumination devices with a reliable light output to treat patients.

**Figure 1:**
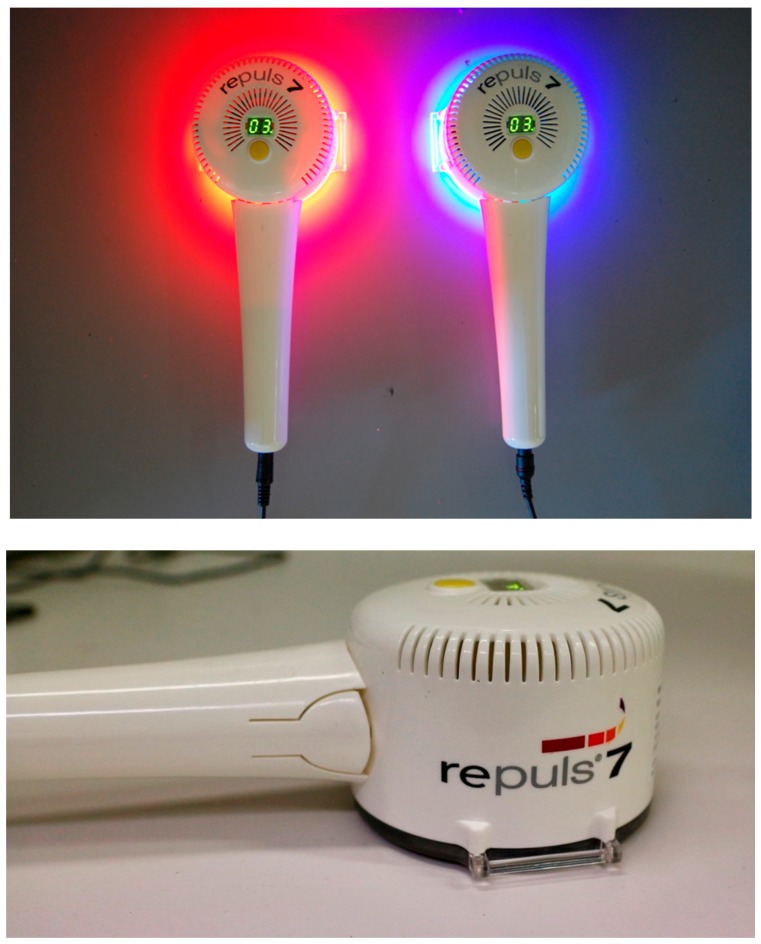
Repuls7PDI-red and -blue light emitting diode (LED) laser illumination device.

In this paper we introduce two light sources tailored for the requirements in PDI, the Repuls7PDI-red and Repuls7PDI-blue (Figure 1). Both are manufactured by Repuls Lichtmedizintechnik GmbH and based on the Repuls7 lamp, which holds approval as a medical device class 2b [13]. The lamps consist of seven high-power LEDs of the latest generation. With its low weight, the device fits well in the hand, and is therefore easy to use. The exposure time can be set individually in three-minute steps from 3–30 min with automatic switch off. A cooling fan prevents heating up of the device, which is required for application in human medicine.

Two different photosensitizers are tested here: The phenothiazinium dye Methylene blue (MB), which is widely used in PDI [4] and which can be photoactivated with the Repuls7PDI-red, and the natural compound Sodium Magnesium chlorophyllin (CHL), which holds approval as food additive E100. This chlorin e6 derivative was photoactivated with both, that is, the Repuls7PDI-blue and Repuls7PDI-red. As model systems for efficiency testing against bacteria, Gram+ *Staphylococcus aureus* and Gram− *Escherichia coli* are used, and the antifungal properties of PDI were proven against *Candida albicans*.

## 2. Results

### 2.1. Irradiance Spectrum of the Repuls7PDI-Red and -Blue

**Figure 2:**
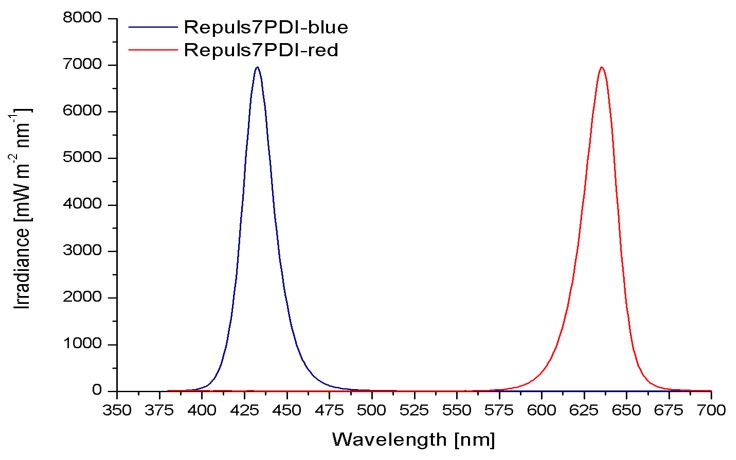
Irradiance spectrum of the Repuls7PDI-blue and the Repuls7PDI-red. The Repuls7PDI-blue shows a distinct irradiance peak at 433 nm (blue line) and the Repuls7PDI-red (red line) peaks at 635 nm.

The Repuls7PDI-red (Figure 2, red line) shows a distinct irradiance peak at 635 nm ± 11.5 nm with an irradiance of 6961.49 mW·cm^−2^ and at full width a half maximum (FWHM) of 23 nm, and for the Repuls7PDI-blue (Figure 2, blue line) at 433 nm ± 10.5 nm (FWHM 21 nm). The irradiance at the wavelength maximum is 6964.98 mW·cm^−2^·nm^−1^.

### 2.2. Spatial Distribution of the Irradiance of the Repuls7PDI-Red and Blue Light Source

**Figure 3:**
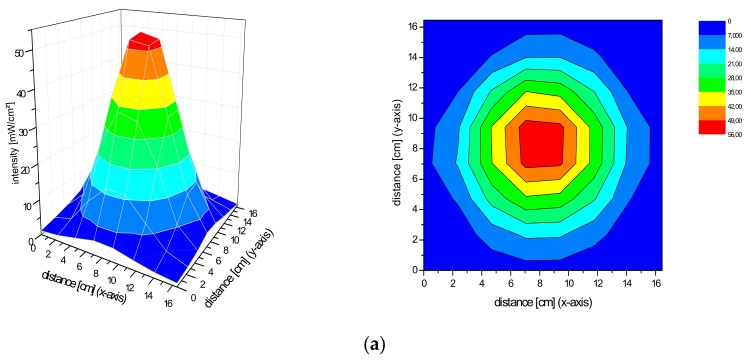

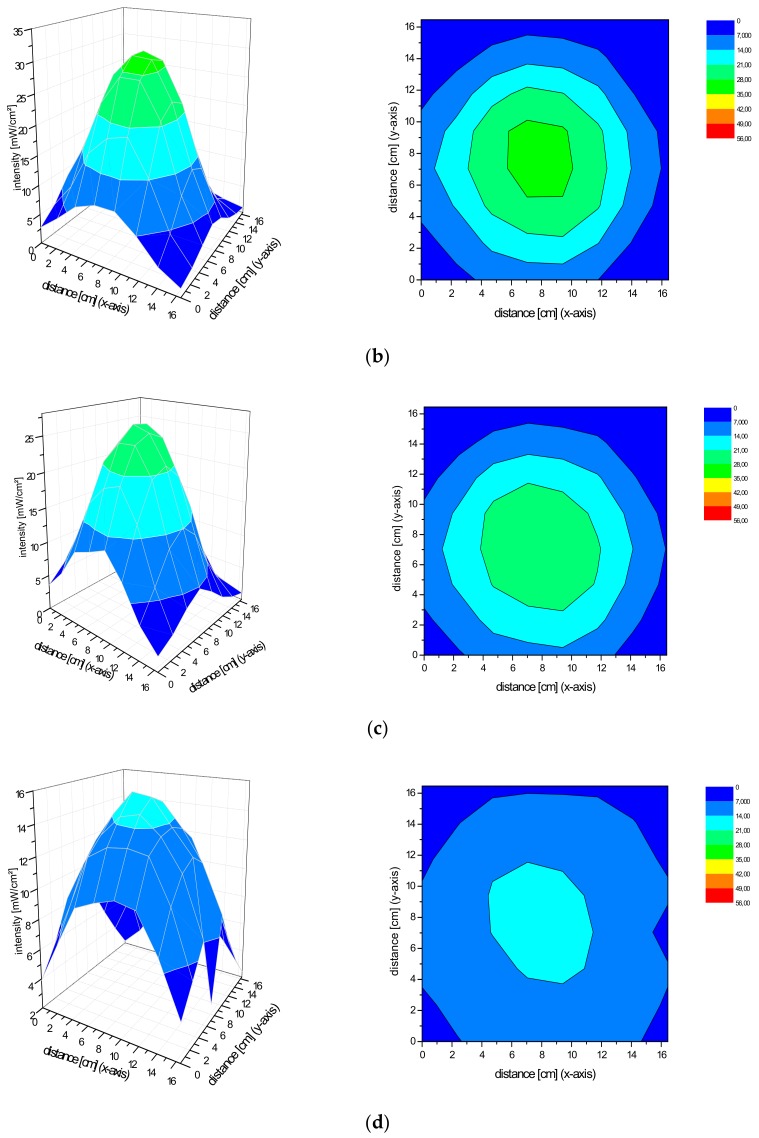
(**a**) Irradiance distribution of the Repuls7PDI-red at a distance of 10 cm. The device was fixed with a tripod with the radiating surface parallel downwards to the measuring plane. (**b**) Irradiance distribution of the Repuls7PDI-red at a distance of 13.5 cm. The device was fixed with a tripod with the radiating surface parallel downwards to the measuring plane. (**c**) Irradiance distribution of the Repuls7PDI-red at a distance of 15 cm. The device was fixed with a tripod with the radiating surface parallel downwards to the measuring plane. (**d**) Irradiance distribution of the Repuls7PDI-red at a distance of 20 cm. The device was fixed with a tripod with the radiating surface parallel downwards to the measuring plane.

**Figure 4:**
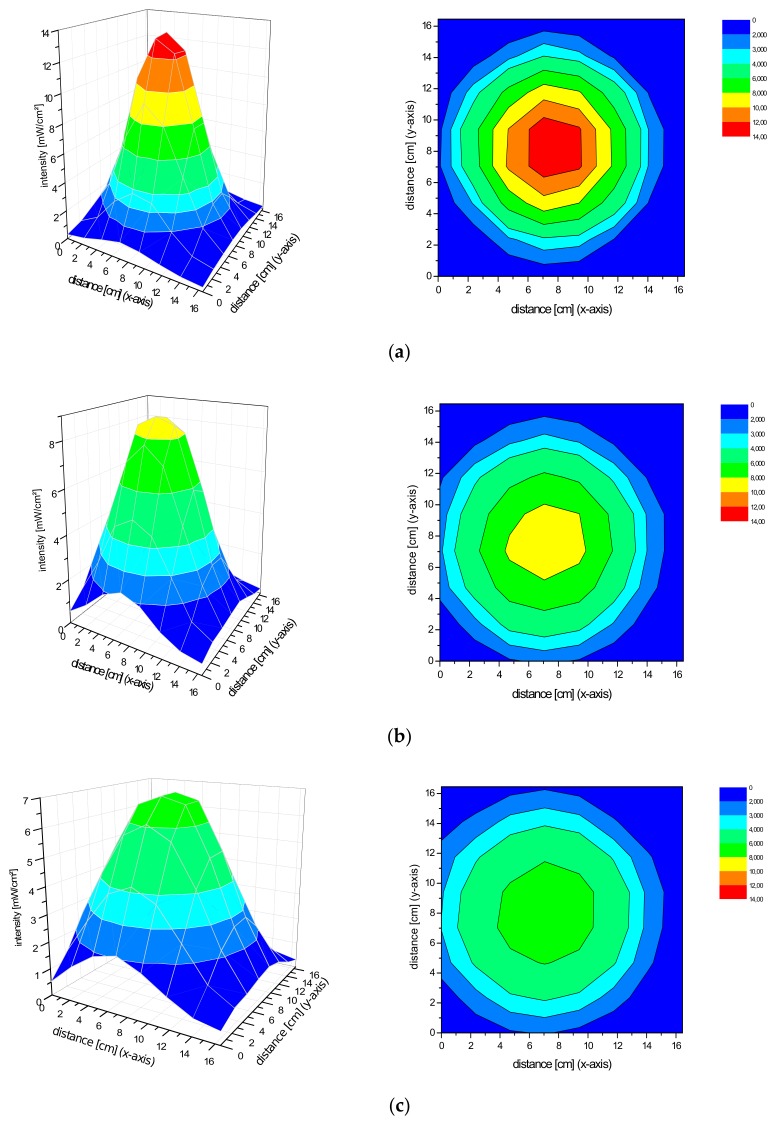

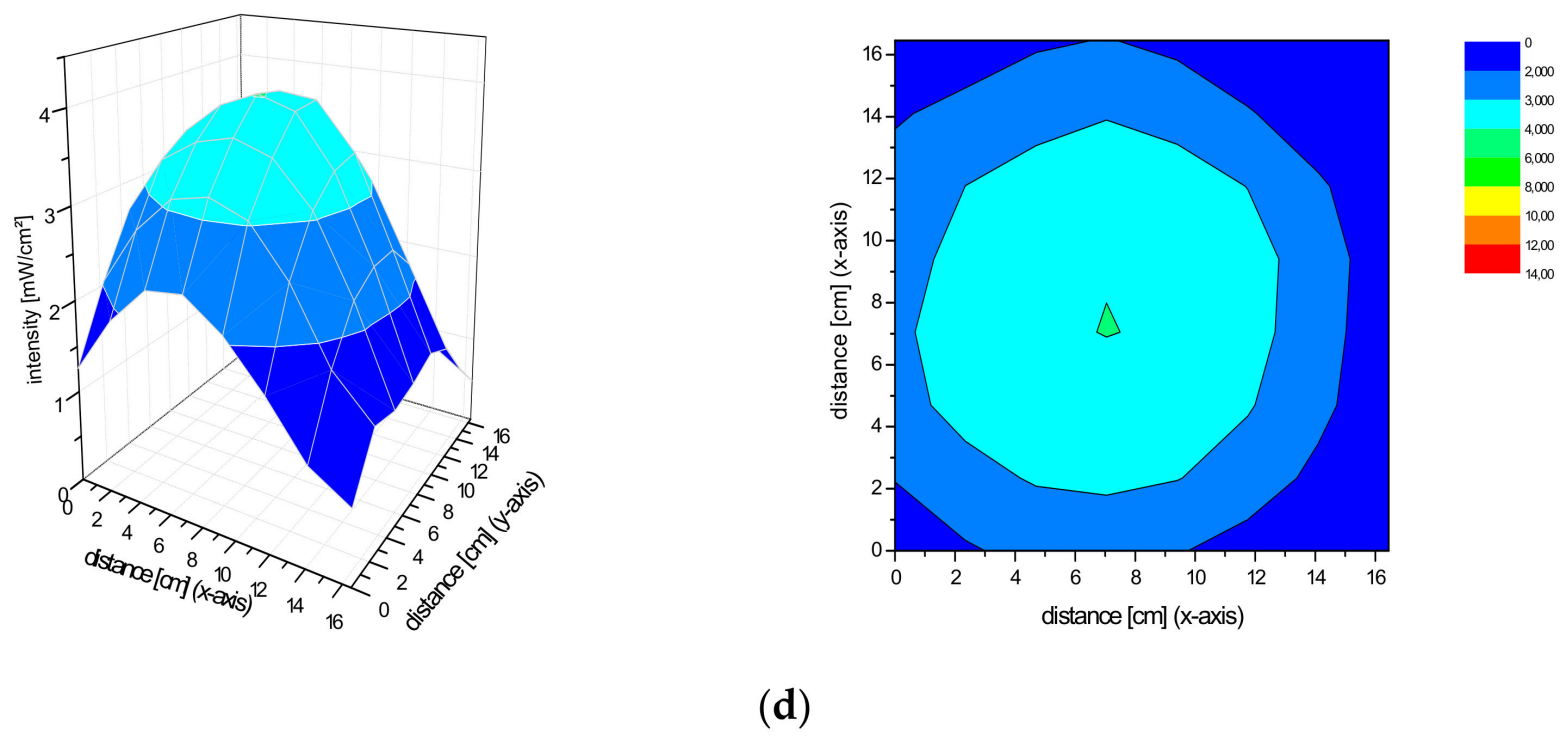
(**a**) Irradiance distribution of the Repuls7PDI-blue at a distance of 10 cm. The device was fixed with a tripod with the radiating surface parallel downwards to the measuring plane. (**b**) Irradiance distribution of the Repuls7PDI-blue at a distance of 13.5 cm. The device was fixed with a tripod with the radiating surface parallel downwards to the measuring plane. (**c**) Irradiance distribution of the Repuls7PDI-blue at a distance of 15 cm. The device was fixed with a tripod with the radiating surface parallel downwards to the measuring plane. (**d**) Irradiance distribution of the Repuls7PDI-blue at a distance of 20 cm. The device was fixed with a tripod with the radiating surface parallel downwards to the measuring plane.

The homogeneity of a light source is defined by a low variation in the spatial distribution of the irradiance. Basically, the Repuls7PDI-red/blue both produce a conical light distribution. The smaller the distance between the diode plane and measuring plane, the higher the maximum irradiance. As the distance increases, so does the size of the homogeneous exposure field, as measured by the standard deviation of the irradiance over the central field (5 cm × 5 cm) in size. For the Repuls7PDI-red at a distance of 10 cm between the lamp and the detector, the average central field irradiance is 40.54 ± 10.76 mW·cm^−2^ (Figure 3a). Upon increase of the distance between the illumination device and detector to 13.5 cm (Figure 3b), 15 cm (Figure 3c) or 20 cm (Figure 3d), the average irradiance in the field of interest drops to 27.64 ± 2.41 mW·cm^−2^, 24.35 ± 1.61 mW·cm^−2^ and 14.60 ± 0.78 mW·cm^−2^, respectively. The relative error—calculated by 100*standard deviation/arithmetic mean—drops from 26.54% at the 10 cm distance to 5.32% at 20 cm. The average intensity of the Repuls7PDI-blue is lower when compared to the Repuls7PDI-red, but this may be compensated by the higher absorption of porphyrins in the Soret-band when compared to the Q-band [14,15]. The average intensities are 10.62 ± 2.34 mW·cm^−2^ at 10 cm (Figure 4a), 7.95 ± 0.68 mW·cm^−2^ at 13.5 cm (Figure 4b), 6.39 ± 0.51 mW·cm^−2^ at 15 cm (Figure 4c) and 3.81 ± 0.18 mW·cm^−2^ at 20 cm (Figure 4d). Once again, with increasing distance between the LED array and the detector, the average intensity drops, but the homogeneity in the 5 cm × 5 cm field of interest increases.

### 2.3. PDI Based on Methylene Blue against E. coli, S. aureus and C. albicans

**Figure 5:**
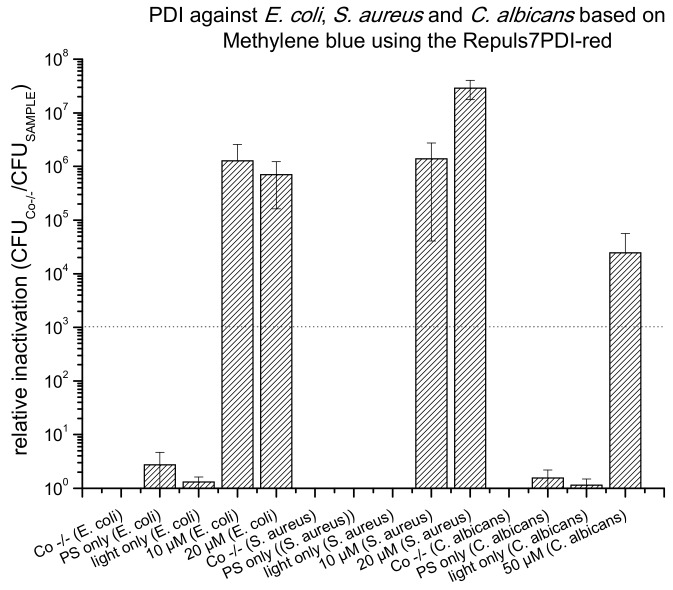
Photodynamic Inactivation of *E. coli*, *S. aureus* and *C. albicans* shown as relative inactivation (CFU_Co −/−_/CFU_sample_). Methylene blue was photoactivated using the Repuls7PDI-red (radiant exposure: 25.6 J·cm^−2^, irradiance: 26.3 mW·cm^−2^). The bars represent the average of three independent biological replicates including the standard deviations as error bars. The black dashed line corresponds to a reduction of 3 log_10_ (99.9% killing efficacy). Three controls were included in this experiment. Co −/−: double negative control; PS only: dark control; and light only: light control.

To prove principle that an effective and reproducible PDI can be performed using the Repuls7PDI-red light source, the photokilling of bacteria and fungi using MB as photoactive compound were performed (Figure 5). After 15 min of incubation with 10 µM MB, the number of viable *E. coli* was reduced by a factor 1.27 × 10^6^ after photoactivation with 25.6 J·cm^−2^ at 635 nm. The same illumination parameters allowed for the photokilling of *S. aureus* (20 µM MB, 15 min incubation period) by 2.90 × 10^7^, 10 µM MB by 1.39 × 10^6^ and 2.45 × 10^4^ of *C. albicans* (50 µM MB, 15 min incubation period). There is no dark toxicity of MB, and no phototoxicity of the light alone (light only).

### 2.4. PDI Based on Chlorophyllin against E. coli, S. aureus and C. albicans

**Figure 6:**
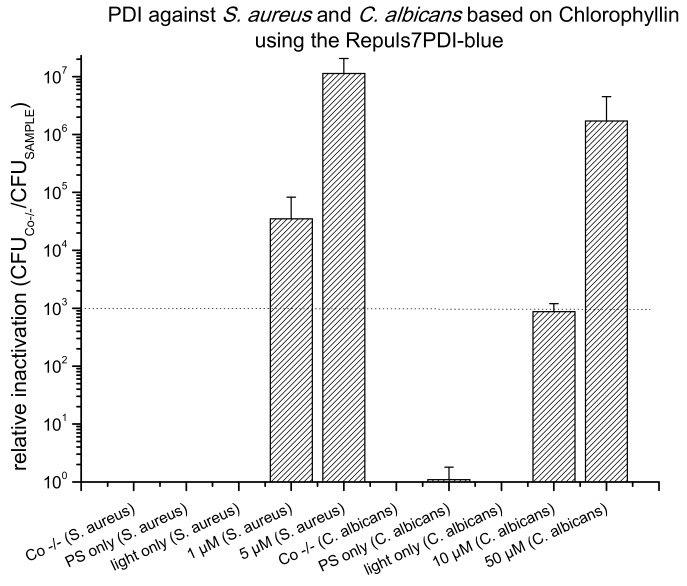
Photodynamic Inactivation of *S. aureus* and *C. albicans* shown as relative inactivation (CFU_Co −/−_/CFU_sample_). Chlorophyllin was photoactivated using the Repuls7PDI-blue (radiant exposure: 6.6 J·cm^−2^, irradiance: 9 mW·cm^−2^). The bars represent the average of three independent biological replicates including the standard deviations as error bars. The black dashed line corresponds to a reduction of 3 log_10_ (99.9% killing efficacy). Three controls were included in this experiment. Co −/−: double negative control; PS only: dark control; and light only: light control.

**Figure 7:**
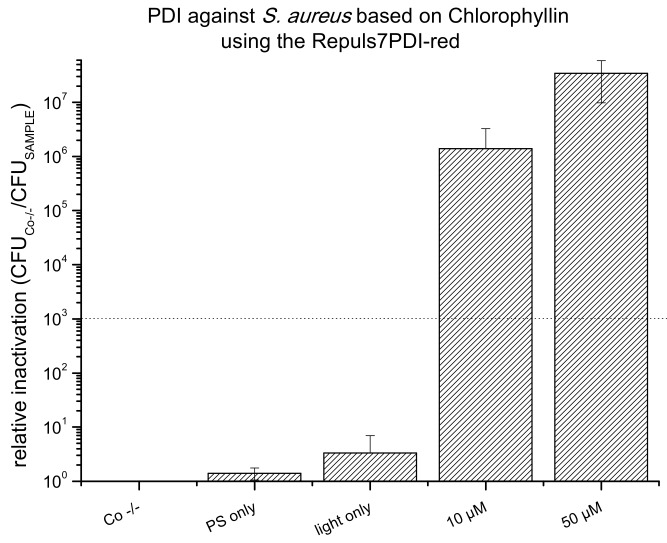
Photodynamic Inactivation of *S. aureus* shown as relative inactivation (CFU_Co −/−_/CFU_sample_). Chlorophyllin was photoactivated using the Repuls7PDI-red (radiant exposure: 25.6 J·cm^−2^, irradiance: 26.3 mW·cm^−2^). The bars represent the average of three independent biological replicates including the standard deviations as error bars. The black dashed line corresponds to a reduction of 3 log_10_ (99.9% killing efficacy). Three controls were included in this experiment. Co −/−: double negative control; PS only: dark control; and light only: light control. The black dashed line corresponds to a reduction of 3 log_10_ units as the criterion for an antibacterial effect.

As a result of their absorption properties, porphyrins can be photoactivated in the blue (Soret band, ~410 nm) or red wavelength range (Q band, ~630–660 nm). Upon activation with the Repuls7PDI-blue (433 nm, illumination period 15 min, radiant exposure 6.6 J·cm^−2^) a PDI using 1 µM CHL killed 7 log_10_ steps of *S. aureus* and 5 µM CHL 4 log_10_ (Figure 6). Photoactivated CHL proved to be very effective against the yeast *C. albicans:* At 10 µM PS concentration an antifungal effect was achieved (3 log_10_ criterion), and with 50 µM CHL a complete eradication could be achieved (more than 6 log_10_ reduction of CFU). Once more, neither the activating light (light only), nor the photoactive compound without illumination showed any cytotoxic effect (Figure 6). Chlorophyllin does not carry a cationic charge, and is therefore not photoeffective against Gram– *E. coli* (data not shown). To test the red-light activation of CHL, *S. aureus* incubated with either 10 or 50 µM CHL was illuminated using the Repuls7PDI-red (25.6 J·cm^−2^), leading to a bacterial kill of 6 log_10_ (10 µM), or below the detection limit (>7 log_10_ at 50 µM, see Figure 7).

## 3. Discussion

Photodynamic Inactivation is a powerful antimicrobial approach, and represents an alternative to the use of conventional antibiotics. As resistant bacteria and fungi can be killed, it will be helpful in the future treatment of diseases caused by microorganisms [16]. Although Photodynamic Inactivation is a daughter of Photodynamic Therapy against cancer, not all concepts of antitumor treatment can be transferred to the antimicrobial application. So for example, photosensitizers are required carry a cationic charge to be effective against Gram− bacteria [17], which is not needed against cancer. On the other hand, the substances used for PDT preferentially absorb red or near infrared light to allow for deep tissue penetration, which is not mandatory for any local treatment of superficial infections by PDI [15,17]. These circumstances have been taken into account by chemists, who tailored new photosensitizers for PDI [18]. Some of these substances have been successfully tested in vitro, and are ready for clinical trials. However, the second component in the photodynamic process, the light source for the activation of the photoactive compound, is still not given the required attention. In PDT, relatively small lesions inside the human body are treated. To achieve sufficient light delivery to the tumor, fiber optics are usually coupled to lasers [19]. Although solid-state lasers are easy-to-use and robust, they still require maintenance and the training of users. In addition, the acquisition costs are considerably high when compared to other light sources. Due to their construction principle, they provide a high intensity at relatively small areas, and widening of the narrow laser beam requires sensitive optics. As a result, for the treatment of (larger) pre-cancers or cancers in dermatology, non-coherent illumination systems are widely used. To avoid unwanted thermal effects, filters are necessary to remove those parts of the spectrum of broadband lamps, which are not needed for the photodynamic process. Most of these illumination devices are technically based on one or a few lamps, which may result in a relatively inhomogeneous spatial light distribution [8]. In addition, the operation hours of these lamps are limited; so, for example, the metal halide lamp of the Waldmann PDT-1200 needs replacement for every 200 h of operation [20].

To overcome the limitations of these light sources, LED-based arrays have entered the stage more than 10 years ago. They provide a high light output with low heat production, emit a narrow wavelength range (usually below ±10 nm of the dominant wavelength), which avoids thermal side effects, and offer a homogenous spatial light distribution if based on many individual LEDs. Moreover, LED lamps are maintenance free and easy to handle [7].

Besides home-build devices, some manufacturers offer LED-based lamps at reasonable pricing (see Table 1). However, based on the literature on hand, none of these holds approval as a medical device.

Both, the Repuls7PDI-red and the Repuls7PDI-blue introduced here represent LED-based instruments tailored for the application in PDI. They are derived from the Repuls7 lamp, which is successfully applied in photobiomodulation [21], and is approved as a medical device class 2b [13]. The Repuls7PDI lamp family features an easy-to-use, concept, where only one button controls all of the functions of this device, with adjustable illumination periods from 3 to 30 min, a cooling fan to prevent overheating, holding straps for easy fixation on extremities and a high and homogenous light output. Given the conical light distribution of the seven LEDs mounted in the Repuls7PDI, the size of the area providing a homogenous light field depends upon the distance between the light source and target, and can be controlled by the operator. Small lesions can therefore be treated at a shorter operation distance and short illumination periods, and larger infected areas will require a greater distance and longer light activation of the photoactive compound.

For proof of the principle of the applicability of the Repuls7PDI, we tested two photosensitizers and three different microbial model systems. Phenothiazinium dyes, such as MB [4], are widely used in PDT, and applications range from the treatment of diabetic leg ulcers [22], of infected burn wounds [23] and antibacterial fabrics [24], to environmental applications such as the treatment of plant pathogens [16]. Our results using red light activation prove the photoantimicrobial activity of MB against all bacteria and fungi employed in this study. Even though the dominant wavelength of the Repuls7PDI-red (635 nm) does not exactly match the absorption maximum of MB (660 nm, [25]), comparable low concentrations (20 µM) allow for very effective killing (>6 log_10_) of both pathogens, *S. aureus* and *E. coli*. The inactivation of *S. aureus* was somewhat lower at 10 µM MB. For photoinactivation of the yeast *C. albicans* 50 µM of MB are required for an antifungal effect. This is not in line with the study of Freire et al. [26], where MB without additives was not photoeffective. Significant cell photokilling could only be triggered by combining 100 μM of MB with 100 mM potassium iodide and laser photoactivation (660 nm/40 J·cm^2^). One possible reason why the experiment in our study was successful with 50 µM of MB against *C. albicans* could be the homogeneity of the Repuls7PDI-red light source. This leads to an evenness and more effective exposure. The use of the combination of MB, potassium iodide and the Repuls7PDI emitter, could probably induce a complete elimination of the microorganisms.

Sodium Magnesium Chlorophyllin is a water-soluble derivative of natural chlorophyll, and holds approval as food additive E140. Due to its excellent biocompatibility, CHL has been successfully applied for the Photodynamic Decontamination of food [27,28], as well as the photodynamic killing of bacterial plant pathogens [5]. As expected, due to the lack of cationic moieties that interact with the cell wall/membrane of Gram– bacteria [6], CHL is not phototoxic against Gram– bacteria without cell-wall permeabilizing agents. Additives such as ethylenediaminetetraacetic acid (EDTA) [29] might help to photokill these bacteria, but these additives have not been tested in the frame of this study. Against *S. aureus* photoactivation at the Soret band of CHL using the Repuls7PDI-blue is effective at considerably lower concentrations when compared to the red light activation of MB: at 1 µM a clear antibacterial effect is induced (>4 log_10_ inactivation), and at 5 µM a complete eradication below the detection limit is achieved (>7 log_10_). This is also valid for antifungal PDI towards *C. albicans*: at 50 µM, CHL achieves an about two orders of magnitude higher phototoxicity. Chlorophyllin may also be activated with red light. However, even though the Repuls7PDI-red provides a higher irradiance (27.6 mW·cm^−2^) than the Repuls7PDI-blue (6.4 mW·cm^−2^), an about 10-fold higher CHL concentration (5 µM at 433 nm, 50 µM at 635 nm) is required to achieve complete cell killing below the detection limit. This is attributed to a much higher absorption of CHL at the Soret band when compared to the Q-band [30].

Taken together, both the Repuls7PDI-red and Repuls7PDI-blue are suitable for Photodynamic Inactivation. They provide high and homogenous light output, an easy-to-use concept and high photokilling against microorganisms using the photoactive compounds tested in this study. Standardized lamps have the potential to allow for better compatibility between individual studies, therefore, development of these is required to push forward PDI. As the conceptual parent illumination device, the Repuls7 is registered as medical device [13], approval of the daughters developed for PDI appears achievable. This, of course, is an important factor to facilitate the translation of PDI into clinical practice, as approved devices are tested for electrical safety, heat emission below the permitted limits and come with a clear and safe operation and cleaning concept.

## 4. Materials and Methods

### 4.1. Measurement of the Irradiance Spectrum and Field Homogeneity of the REPULS7PDI-Red and -Blue

The irradiance spectrum of the lamps was recorded using a LI-180 Spectrometer (LI-COR, Lincoln, NE, USA). The measurement was performed at a distance of 16 cm, and was not changed during the entire detection and the spectra were recorded in automatic mode.

The intensity distribution of the Repuls7PDI-red and -blue light source and the determination of the homogeneity were measured by using a LI-189 light meter equipped with a PY pyranometer detector (LI-COR, Lincoln, NE, USA). A grid with circles of the same size was created for the measurement, and the survey was carried out in a darkened room. The pyranometer detector was at the selected distance to the lamp during the entire measurement. The homogeneity was calculated for the center illumination field (5 × 5 cm) using the mean value and standard deviation within this area.

### 4.2. Preparation of Stock Solutions

Stock solutions of the PSs were obtained by dissolving Chlorophyllin (Magnesium Chlorophyllin sodium salt, Carl Roth GmbH + Co. KG, Karlsruhe, Germany) or Methylene blue (Roth) in ultra pure water to a final concentration of 10 mM. The stocks were stored in the dark at −20 °C until usage.

### 4.3. Bacterial Culture

Gram(+) *Staphylococcus aureus* (ATCC 25923) and Gram(–) *Escherichia coli* (ATCC 25922) were grown in 20 mL medium containing 30 g L^−1^ Todd-Hewitt-Bouillon (Roth) and 3 g L^−1^ yeast extract (Sigma-Aldrich Chemie GmbH, Steinheim, Germany) at 37 °C overnight under constant agitation at 200 rpm on a shaking incubator (MaxQ 4450, Thermo Scientific, Marietta, OH, USA). Pathogenic yeast *Candida albicans* (Mya 273) was grown in 20 mL medium containing 32 g L^−1^ Peptone Casein (Sigma-Aldrich), 20 g L^−1^ yeast extract (Sigma-Aldrich), 5 g L^−1^ sodium chloride (VWR International, Vienna, Austria) and 5 mM sodium hydroxide (Fluka Analytical, Munich, Germany) at 37 °C overnight under constant agitation at 200 rpm on a shaking incubator (MaxQ 4450, Thermo Scientific, Marietta, OH, USA).

### 4.4. PDI Against Gram(+) S. aureus, Gram(−) E. coli and the Yeast C. albicans

The bacterial suspensions were centrifuged at 830 rcf (centrifuge 5417R, Eppendorf, Hamburg, Germany) for 3 min. The supernatant was carefully removed and the pellets were reconstituted in DPBS (Dulbecco’s Phosphate Buffered Saline, Sigma-Aldrich) containing 1 µM, 5 µM, 10 µM, 20 µM, 50 µM or 100 µM of the PS. All samples were incubated for 15 min in the dark. Following incubation, the samples were transferred into 24-well plates, and subsequently illuminated from above (13.5 cm), using one of the two LED-based lamps (Repuls7PDI-blue light 433 nm/Repuls7PDI-red light 635 nm). The irradiance of the Repuls7PDI-red (26.3 mW·cm^−2^, radiant exposure: 25.6 J·cm^2^) and the Repuls7PDI-blue (9 mW·cm^−2^, radiant exposure: 6.6 J·cm^2^) was measured using a LI-189 light meter equipped with a PY pyranometer detector (LI-COR, Lincoln, NE, USA). Each experiment included three controls: Co −/− (no PS, no light), light only (no PS, light) and PS only (PS at the maximal concentration, no light). The treated samples were serially diluted in DPBS and plated on petri dishes of the corresponding media containing 1.5% Agar–Agar (Kobe I, Carl Roth). The evaluation was done by counting of the CFU after one day of incubation at 37 °C. All experiments were repeated at least three times.

### 4.5. Data Analysis

The relative inactivation was calculated by dividing the CFU of the double negative control by the CFU of the sample for each biological replicate, as described in Glueck et al. [31]. In the case of a total eradication of viable bacteria (CFU = 0), the CFU of the Co −/− was divided by one. All values represent the mean and standard deviation of at least three biological replicates. The black dashed line in the graphs corresponds to a reduction of 3 log_10_ as criterion for an antibacterial effect.

## Figures and Tables

**Table 1:** Commercially-available LED-based lamps.

Manufacturer	Model	Wavelength(s) Emitted	Weblink
OmniLux	OminLux blueTM	415 ± 10 nm	https://omniluxled.com/products/omnilux-blue/
HUBEI YJT Technology	Aesthetic Phototherapy Lamp	Red light: 630 nm ± 10 nm Blue light: 465 nm ± 10 nm	https://www.medicalexpo.com/prod/hubei-yjt-technology/product-126671-924289.html
Poly	Clear	415 nm	https://www.mypolyled.com/clear/
Poly	Rejuv	433 nm	https://www.mypolyled.com/rejuv/
BIOPTRON	Bioptron Medall	630 nm	https://bioptron.at/index.php/bioptron-produkte.html
Molteni Therapeutics	VULNOLIGHT^®^	630 nm	http://www.moltenitherapeutics.it/products/vulnolight®.aspx
Biofrontera	BF-RHODOLED^®^	635 nm	https://www.biofrontera.com/de/produkte.html
Theralase^®^	TLC-1000	660 nm	https://theralase.com/tlc-1000/

## Abbreviations

*C. albicans*, *Candida albicans*; CFU, colony forming unit(s); DPBS, Dulbecco’s phosphate buffered saline; *E. coli*, *Escherichia coli*; PDI, Photodynamic Inactivation; PS, photosensitiser; ROS, reactive oxygen species; *S. aureus*, *Staphylococcus aureus*; FWHM, Full Width at Half Maximum; CHL, Sodium Magnesium Chlorophyllin; MB, Methylene blue.

## References

[1] (2019). Antimicrobial Resistance Home Page. http://www.who.int/en/news-room/fact-sheets/detail/antimicrobial-resistance.

[2] Boucher H.W., Talbot G.H., Bradley J.S., Edwards J.E., Gilbert D., Rice L.B., Scheld M., Spellberg B., Bartlett J. (2009). Bad bugs, no drugs: No ESKAPE! An update from the Infectious Diseases Society of America. Clin. Infect. Dis..

[3] Plaetzer K., Krammer B., Berlanda J., Berr F., Kiesslich T. (2009). Photophysics and photochemistry of photodynamic therapy: Fundamental aspects. Lasers Med. Sci..

[4] Ghorbani J., Rahban D., Aghamiri S., Teymouri A., Bahador A. (2018). Photosensitizers in antibacterial photodynamic therapy: An overview. Laser Ther..

[5] Glueck M., Hamminger C., Fefer M., Liu J., Plaetzer K. (2019). Save the crop: Photodynamic Inactivation of plant pathogens I: Bacteria. Photochem. Photobiol. Sci..

[6] Krüger M., Richter P., Strauch S.M., Nasir A., Burkovski A., Antunes C.A., Meißgeier T., Schlücker E., Schwab S., Lebert M. (2019). What an Escherichia coli Mutant Can Teach Us About the Antibacterial Effect of Chlorophyllin. Microorganisms.

[7] Pieslinger A., Plaetzer K., Oberdanner C.B., Berlanda J., Mair H., Krammer B., Kiesslich T. (2006). Characterization of a simple and homogeneous irradiation device based on light-emitting diodes: A possible low-cost supplement to conventional light sources for photodynamic treatment. Med. Laser Appl..

[8] Crosbie J., Winser K., Collins P. (2002). Mapping the light field of the Waldmann PDT 1200 lamp: Potential for wide-field low light irradiance aminolevulinic acid photodynamic therapy. Photochem. Photobiol..

[9] King A. (2014). Antibiotic Resistance Will Kill 300 Million People by 2050. https://www.scientificamerican.com/article/antibiotic-resistance-will-kill-300-million-people-by-2050/.

[10] Harrison J.W., Svec T.A. (1998). The beginning of the end of the antibiotic era? Part II. Proposed solutions to antibiotic abuse. Quintessence Int..

[11] Yilmaz A., Ozkiraz S., Akcan A.B., Canpolat M. (2015). Low-cost Home-use Light-emitting-diode Phototherapy as an alternative to Conventional Methods. J. Trop. Pediatr..

[12] Peloi L.S., Soares R.R., Biondo C.E., Souza V.R., Hioka N., Kimura E. (2008). Photodynamic effect of light-emitting diode light on cell growth inhibition induced by methylene blue. J. Biosci..

[13] Repuls ist Nach den Neuesten Anforderungen der MDD (Medizinprodukterichtlinie) Zugelassen. https://www.repuls.at/.

[14] Stockett M.H., Musbat L., Kjær C., Houmøller J., Toker Y., Rubio A., Milne B.F., Nielsen S.B. (2015). The Soret absorption band of isolated chlorophyll a and b tagged with quaternary ammonium ions. Phys. Chem. Chem. Phys..

[15] Cieplik F., Deng D., Crielaard W., Buchalla W., Hellwig E., Al-Ahmad A., Maisch T. (2018). Antimicrobial photodynamic therapy—What we know and what we don’t. Crit. Rev. Microbiol..

[16] Wainwright M., Maisch T., Nonell S., Plaetzer K., Almeida A., Tegos G.P., Hamblin M.R. (2017). Photoantimicrobials-are we afraid of the light?. Lancet Infect. Dis..

[17] Tortik N., Steinbacher P., Maisch T., Spaeth A., Plaetzer K. (2016). A comparative study on the antibacterial photodynamic efficiency of a curcumin derivative and a formulation on a porcine skin model. Photochem. Photobiol. Sci..

[18] Abrahamse H., Hamblin M.R. (2016). New photosensitizers for photodynamic therapy. Biochem. J..

[19] Nguyen K., Khachemoune A. (2019). An update on topical photodynamic therapy for clinical dermatologists. J. Dermatol. Treat..

[20] (1997). Waldmann PDT 1200 Instruction for Use Manual.

[21] Winter R., Dungel P., Reischies F.M.J., Rohringer S., Slezak P., Smolle C., Spendel S., Kamolz L.P., Ghaffari-Tabrizi-Wizsy N., Schicho K. (2018). Photobiomodulation (PBM) promotes angiogenesis in-vitro and in chick embryo chorioallantoic membrane model. Sci. Rep..

[22] Tardivo J.P., Serrano R., Zimmermann L.M., Matos L.L., Baptista M.S., Pinhal M.A.S., Atallah Á.N. (2017). Is surgical debridement necessary in the diabetic foot treated with photodynamic therapy?. Diabet. Foot Ankle.

[23] Sperandio F.F., Huang Y.-Y., Hamblin M.R. (2013). Antimicrobial photodynamic therapy to kill Gram-negative bacteria. Recent Patents on Anti-Infective Drug Discovery.

[24] Teli M.D., Sheikh J. (2012). Antibacterial and acid and cationic dyeable bamboo cellulose (rayon) fabric on grafting. Carbohydr. Polym..

[25] Gollmer A., Felgenträger A., Bäumler W., Maisch T., Späth A. (2015). A novel set of symmetric methylene blue derivatives exhibits effective bacteria photokilling—A structure-response study. Photochem. Photobiol. Sci..

[26] Freire F., Ferraresi C., Jorge A.O.C., Hamblin M.R. (2016). Photodynamic therapy of oral Candida infection in a mouse model. J. Photochem. Photobiol. B Biol..

[27] Zudyte B., Luksiene Z. (2019). Toward better microbial safety of wheat sprouts: Chlorophyllin-based photosensitization of seeds. Photochem. Photobiol. Sci..

[28] Paskeviciute E., Zudyte B., Luksiene Z. (2019). Innovative Nonthermal Technologies: Chlorophyllin and Visible Light Significantly Reduce Microbial Load on Basil. Food Technol. Biotechnol..

[29] George S., Hamblin M.R., Kishen A. (2009). Uptake pathways of anionic and cationic photosensitizers into bacteria. Photochem. Photobiol. Sci..

[30] Seely G.R., Jensen R.G. (1965). Effect of solvent on the spectrum of chlorophyll. Spectrochim. Acta.

[31] Glueck M., Schamberger B., Eckl P., Plaetzer K. (2017). New horizons in microbiological food safety: Photodynamic Decontamination based on a curcumin derivative. Photochem. Photobiol. Sci..

